# The proximal segment of the embryonic outflow (conus) does not participate in aortic vestibule development

**DOI:** 10.1371/journal.pone.0209930

**Published:** 2018-12-31

**Authors:** Roberto Lazzarini, Luis Enrique Gómez-Quiroz, Humberto González-Márquez, Laura Villavicencio-Guzmán, Marcela Salazar-García, Concepción Sánchez-Gómez

**Affiliations:** 1 Doctorado en Ciencias Biológicas y de la Salud, Universidad Autónoma Metropolitana, Ciudad de México, México; 2 Departamento Ciencias de la Salud, Universidad Autónoma Metropolitana Iztapalapa, Ciudad de México, México; 3 Laboratorio de Investigación en Biología del Desarrollo y Teratogénesis Experimental, Hospital Infantil de México, Federico Gómez, Ciudad de México, México; New York Medical College, UNITED STATES

## Abstract

**Objective:**

There is no consensus on the embryonic components or morphogenetic processes involved in mature ventricular outflow tract development. Our goal was to use *in vivo* labelling to investigate the prospective fate of the myocardium of each conal wall. The conal and atrioventricular cushion mesenchyme changes during transformation into mature structures and their role in apoptosis were also investigated.

**Methods:**

Plastic labels were placed at the cephalic and caudal conal limits of chicken embryo hearts (stage 22HH) and traced up to stage 36HH. Histological analyses, scanning electron microscopy and apoptotic detection using Lysotracker-Red were performed. The conal longitudinal length and medial displacement were registered. Muscle myosin was identified by immunofluorescence.

**Results:**

Labels positioned in the myocardium of each conal wall moved to the right ventricle (RV), shifting from the arterial subvalvular myocardial zone to the apex. No labels were found in the aortic vestibule. At stage 22HH, the conus was a tubular structure composed of myocardium and endocardium with scarce mesenchyme. The dorso-left conal myocardial wall gradually lost continuity and the free ends separated, while the myocardium was distributed to the RV free wall (24HH-28HH). At stage 22HH, conal crests were not observed, but they were apparent at the dorsal zone of the conus at stage 26HH; towards stage 30HH, they fused to form the supraventricular crest, and the pulmonary infundibulum was evident. The ventro-superior cushion of the AV canal was reorganized into the fibrous and muscular structures lined the aortic vestibule.

**Conclusions:**

The posterior conus is an erroneous concept. The conal myocardium is reorganized in the free wall of the RV. Internally, the conal lumen is transformed into the pulmonary infundibulum. The aortic vestibule is formed from the ventro-superior cushion of the AV canal. Thus, the ventricular outflow tracts have different embryonic origins.

## Introduction

**Fig 1:**
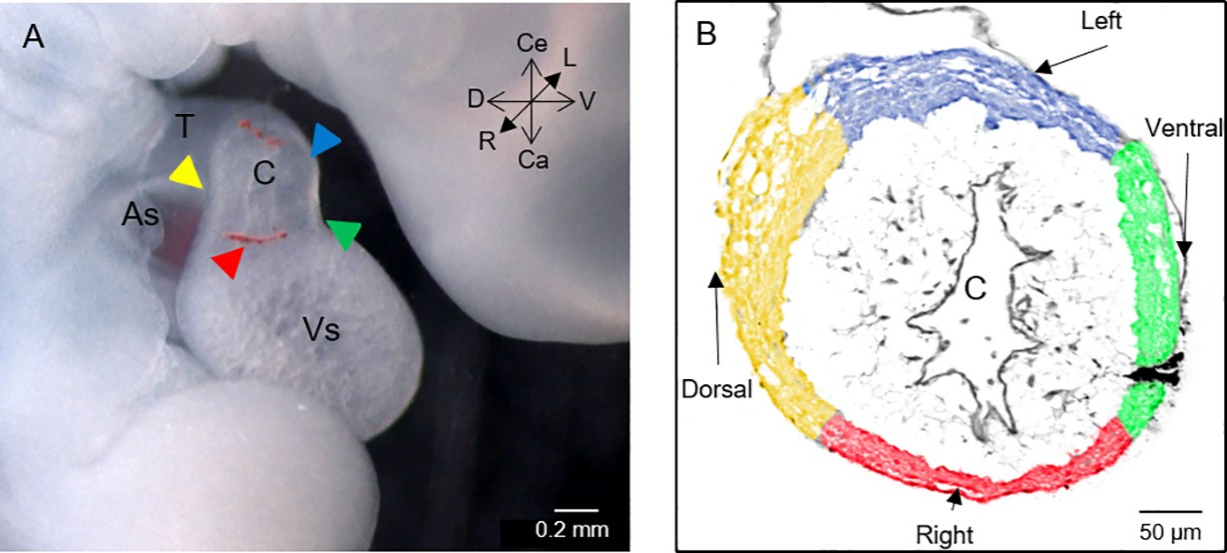
Spatial situation of the myocardial conal walls. (A) Right sagittal view of the cephalic portion of a stage 22HH chicken embryo showing the different regions that make up the heart and the location of the conal walls in the organ in situ. Arrowheads: yellow = dorsal wall; green = ventral wall; red = right wall; blue = left wall. (B) Cross-section of the middle zone of the conus at stage 22HH, haematoxylin-eosin stain. Digitally, colours were impressed to the conal walls myocardium as described in section A. Observe a black label immersed in the myocardium of the ventral conal wall. Abbreviations: As = atrial segment; C = conus; Ca = caudal; Ce = cephalic; D = dorsal; L = left; R = right; T = truncus; V = ventral; Vs = ventricular segment.

It is generally accepted at present that the ventricular outflow tracts of the postnatal tetracavitary heart consist of three well-defined anatomical regions: arterial trunks (pulmonary and aortic trunks), arterial valves (valvar leaflets and their supporting annulus) and subvalvular intracardiac ventricular outflow tracts (pulmonary infundibulum and aortic vestibule) [1]. In agreement with this idea, it has been pointed out that the embryonic outflow also consists of three segments: distal, intermediate and proximal [2]. The distal segment corresponds to the aortic sac with a vascular composition, while the intermediate and proximal segments of the developing outflow comprise the truncus and conus, both of which are covered by a myocardial sleeve. Additionally, the concept of the first (FHF) and second (SHF) heart fields has recently been developed [3–5]. The straight heart tube represents the FHF, while the SHF originates the latter, which gradually converges into the heart tube during the torsion and looping process and is the cellular source from which the embryonic outflow, inflow and atria are derived. Despite this new anatomical approach of the embryonic and postnatal heart, and the discoveries in the two heart fields, there is still no consensus on the embryonic components involved in the genesis of the intrapericardial arterial trunks. Additionally, the information about each element that forms the mature intracardiac ventricular outflow tracts is controversial. There is also no agreement on the morphogenetic processes involved in the development of each of these cardiac structures. Most classic authors of cardiac embryology indicate that the truncus is involved in all [6–15] or only in a part of the vascular extension of the great arteries, including the aortic and pulmonary valves formation [2, 16]. However, in 2005 we concluded that the truncus is involved only in the development of the pulmonary and aortic insertion annuls and valves, but it does not form the entire vascular region of the great arteries, which is derived from the aortic sac [17]. This statement was supported by at least two findings in the chick embryo heart: 1) The ultrastructural and experimental evidences of myocardial cell differentiation in to connective tissue in the truncus [18]. 2) The high incidence of persistent truncus arteriosus associated with anomalies of the aortic arches resulted from the neural crest cells between the third and right fourth aortic arches region depleted [19, 20]. Regarding the embryogenesis of the ventricular outflow tracts, it has been widely reported that the conus decreases its longitudinal dimensions during developmental progression, and its final anatomical representation in the adult heart is limited to the subvalvular area of the intracardiac RV infundibulum [8, 9, 21]. Additional classical and current descriptive studies mention that the conus is initially a tubular structure, with a smooth myocardial wall, without trabeculae, and with a lumen lined circumferentially by cardiac jelly, covered by endocardium. Later, in the advanced loop stage, endocardial ridges develop inside the intermediate and proximal segments of the developing outflow via the endothelial to mesenchymal transformation process [22]. It has also been reported that the right and left endocardial crest divide the conus into an anterior and posterior conduit. The anterior conus will transform into the pulmonary infundibulum (located between the free edge of the supraventricular crest and the pulmonary valve supporting annulus). In contrast, the posterior conus will form the aortic vestibule (located between the mitral-aortic fibrous continuity and the aortic valve supporting the annulus) [6, 7, 23, 24]. In this context, classic *in vivo* labelling studies in the chick embryo heart by De la Cruz et al [24] demonstrated that the conus is fully developed in the advanced loop stage (Stage 22HH). The conus-ventricular limit was described as the transition zone between a smooth conal region, which contrasts with a ventricular trabecular zone. And the boundary between the conus and the truncus was considered the angular junction between the proximal caudo-cephalic and the distal ventro-dorsal segments of the heart tube (Fig 1A).

The differential arrangement of the right and left endocardial conal crests, contrasting with the upper and lower endocardial truncal crests, was further recognized to facilitate the distinction between the conus and the truncus. This description of the chick embryonic heart at stage 22HH by De la Cruz et al [24] is similar to the “dog-leg bend” described in the mouse heart of embryos with 42 somites by Anderson and cols [15]. The de la Cruz delimitation of the anterior conus by differential labelling with a gelatin-Indian ink mixture and tracing of its precise fate throughout the mature heart has confirmed that the anterior conus provides the origin for the right ventricular outflow tract and inferred that the posterior conus will form the aortic vestibule [24]. However, recent studies utilizing cell tracing with a fluorescent dye in the chicken embryo have led to the conclusion that the embryonic outflow forms both the trabecular free wall and the infundibulum of the right ventricle [25].

The idea of the fate of the posterior conus as a precursor of the aortic vestibule has been maintained until the present. However, in the 1980s, two groups of researchers labelled the ventro-superior cushion of the atrioventricular canal (AV canal) in the chicken embryo heart [26, 27]. These researchers found that this cushion was involved in the formation of the fibrous (mitroaortic continuity) and muscular walls of the aortic vestibule, but in no case was a convincing explanation provided for the role of the posterior conus.

Most classic authors point out in humans [9, 21] and birds [13, 24] that the conus undergoes longitudinal reduction; in fact, it has been suggested that the conus completely disappears [10, 12, 13, 23]. Moreover, Watanabe et al by viral transfection of the chicken embryo heart, reported reduction of the cono-truncus myocardium by apoptosis during transformation of the embryonic outflow into the mature anatomical structures [28, 29]. However, they did not mention whether apoptosis was present in the conus, truncus or both regions. Additionally, in murine animal models, the right ventricle (RV) is now known to develop completely from the second heart field [30], which has been identified in embryonic stages as the cono-truncus and a part of the pharyngeal mesoderm [31, 32]. This new information raises questions concerning the fate of the conus as a precursor of both ventricular outflow tracts. Due to all these controversies, by *in vivo* labelling of the chicken embryo heart and histological analysis, we aimed to investigate the reordering of the myocardium of each conal wall and the changes in the mesenchymal tissue of the conus and the AV cushions during their transformation into mature structures. The role of apoptosis was also investigated. We demonstrated that the conus was not divided in two conducts, nor did it display apoptosis. Rather, the conal myocardium participates in the formation of the right ventricle free wall, including the pulmonary infundibulum. Additionally, we identified the ventro-superior cushion of the AV canal as the embryonic precursor of the aortic vestibule.

## Materials and methods

### Nomenclature

In this work, we used the classic conus and truncus nomenclature because although it is obsolete among embryologists, it is still used in the field of paediatric cardiology and pathology. Additionally, one of the basic principles of anatomical states is that all structures within the body should be described as observed in the anatomical position of the subject facing the viewer [33]. In accordance with this principle, the walls of the proximal segment of the embryonic outflow classically known as the conus are named based on their relative positions within the embryo, following the axis symmetry but not their supposed prospective fate as is customary. Thus, we designated right and left conal walls as the classically denoted anterior and posterior conal walls, while the conal walls conventionally considered right and left in this paper were designated as dorsal and ventral walls, respectively (Fig 1).

### Embryos

Fertilized Bovans chicken eggs were obtained from the local poultry farm ALPES (Puebla, México). The eggs were incubated at 37.8°C and 60% relative humidity until they reached stage 22HH [34]. The eggshells were disinfected with 70% alcohol and then windowed to stage the embryos. Having exposed the heart by dissection of the allantoidal and pericardial membranes, the myocardial conal walls were differentially labelled based on the description by de la Cruz et al [24]. The animal use protocols and study procedures were based strictly on the Mexican Official Guidelines (NOM-062-ZOO-1999). In addition, the research, ethics, and biosafety Children's Hospital of Mexico Federico Gomez committees approved this project (HIM-2013-060).

### Plastic labels preparation

Plastic labels were make with a gelatine-activated charcoal mixture with some modifications of the Seichert technique [35]. With this purpose, thin (≤10 μM in diameter) and long (10 cm) filaments were prepared by heating to red hot and extensively stretching a glass rod. India ink (0.1 mL), an aqueous pure 5% gelatine mixture (1 mL) and activated charcoal (0.17 g) were mixed in an Eppendorf tube and gently stirred in warm water (70°C) for 3 min. The liquid mixture was used to varnish the 10 μM glass filaments. Once the mixture of black coloured gelatine had solidified, the varnished filaments were stored in a sterile petri dish under refrigeration. To label the embryonic chicken hearts *in vivo*, small black coloured glass fragments (0.5 mm) were cut and inserted into the previously chosen tissue.

### Labelling experiments

Embryos at stage 22HH, when the conus completes development and the truncus can be certainty identified, were randomly separated into four groups. **GI**. To determine the prospective fate of the classically designated anterior conus, a fine glass filament covered with a gelatine-activated charcoal mixture was inserted for 15 seconds in the myocardium at the boundary between the smooth conal region and the ventricular trabeculated zone. Another filament was inserted at the angular junction between the conus and the truncus. The filaments were subsequently drawn out, leaving a dark tattoo immersed in the myocardium. To place the labels, a 1 cm^2^ window was opened in the egg shell. The vitelline and pericardial membranes were immediately dissected to expose the heart. The prospective fate of the myocardium of the remaining conal walls was determined by placing a label into the myocardium in the middle area of the conus (Stage 22HH) with the following distribution: **GII.** At the ventral conal wall. **GIII.** At the dorsal conal wall **GIV**. At the left conal wall. After labelling, the embryos in the four groups were photographed *in ovo* using a Carl Zeiss stereomicroscope Lumar V12 and a digital camera Axiocam MRC (Carl Zeiss, Germany). Once the window of the shell was covered with Parafilm, the eggs were returned to the incubator and maintained at 37.8°C and 90% relative humidity to acquire embryos at various representative stages of the process of cardiac septation (24-36HH). The labelling procedure causes mortality, and therefore only those embryos that showed a normal morphology and heart with no apparent defects were selected. Forty normal GI hearts from each stage (24–36HH) and twenty GII–GIV hearts from each stage were counted to identify the myocardial conal fate and histological analysis. Additionally, GI embryos were assessed using scanning electron microscopy, morphometric, apoptosis and immunofluorescence analyses. The total hearts acquired at each stage were: GI = 45, GII–GIV = 30 (Table 1).

**Table 1:** Embryonic hearts acquired and number of hearts used for each stage.

Conal Wall labelled	HH Stage	Myocardial conal fate	Histological analysis	Morphometric analysis		Apoptosis analysis	IF Analysis
				Conus Length	Conal Rotation		
GI Right GII Ventral GIII Dorsal GIVLeft	22HH	n = 30	n = 6	n = 20+	n = 20+	n = 6*	n = 6*
	24HH	n = 30	n = 6	n = 20+	n = 20+	n = 6*	n = 6*
	26HH	n = 30	n = 6	n = 20+	n = 20+	n = 6*	n = 6*
	28HH	n = 30	n = 6	n = 20+	n = 20+	n = 6*	n = 6*
	30HH	n = 30	n = 6	n = 20+	n = 20+	n = 6*	n = 6*
	32HH	n = 30	n = 6	n = 20+	n = 20+	n = 6*	n = 6*
	36HH	n = 30	n = 6	n = 20+	n = 20+	n = 6*	n = 6*

Total hearts acquired: GI = 45 at each stage, GII–GIV = 30 at each stage. + Captured images from GI labelled hearts.

* Fresh GI labelled hearts.

### Prospective fate of the myocardial wall of the conus

Mature labelled hearts (stage 36HH) that had completed cardiac septation, great vessel branching, and valve leaflet development were photographed using the same equipment as at the beginning of the experiments (n = 30 in each group). Photographs were used to identify the final location of the labels, define the prospective fate of each myocardial wall of the conus and design a destination map of the conal walls.

### Histological procedures

Six representative labelled fixed hearts at each stage (24-36HH) from GI-GIV previously photographed were dehydrated through a graded series of alcohols to 100%. The hearts were cleared in xylene (Sigma, USA) and embedded in Paraplast wax (Tissue-Tek, USA) to obtain 5 μm serial transverse sections. The sections were stained with haematoxylin and eosin (H&E) to investigate the topographical changes in the conus and the AV canal. A description of the conal myocardium and mesenchymal tissue at the conus and AV cushions was also provided.

### Morphometric analysis

To define the conus length changes and the conus displacement during cardiac septation (stages 22- 36HH), photographs of GI hearts in the frontal view at stages 22- 36HH were used. In the first case (conal length), the distance between the central zone of initially compact black tattoo of the plastic label to the half zone of the dispersed black sports immersed in the myocardial ventricular tissue were measured. In the second case (conus displacement), the opening of the angle between the middle line of the embryo (neural tube) and the sagittal line drawn up throughout the developing conus was registered, using the ImageJ programme (n = 20 captured images at each stage).

### Statistical analysis

For conus length changes and displacement values, data are present as means. Analyses were performed using the Student’s test and ANOVA with Prism software. Differences were considered to be significant at 𝑝 ≤ 0.01.

### Scanning electron microscopy

Ten embryonic GI hearts at different stages (22-36HH) previously fixed and photographed were used for scanning electron microscopy analysis. A group of this hearts (n = 5) was dissected in a transversal plane at the level of the base of the heart to observe the lumen of the conus and the AV canal. In both cases (dissected and complete hearts), the samples were dehydrated and desiccated under liquid CO_2_ with a critical-point drying apparatus Samdri 789A (Tousimins Research Co., MD, USA) and sputter-coated with 350 nm gold in a Denton Vacuum Desk 1A apparatus (Cherry Hill Industrial Centre, NJ, USA). Photographs were obtained using a JEOL Scanning Electron Microscope JSM 5300 (JEOL, Tokyo, Japan) at 15 kV and at different magnifications. Photographs acquired were used to illustrate the gradual displacement of the conus from its original right position to a definitive ventral position, previously measured in the GI hearts captured images.

### Apoptotic pattern at the embryonic outflow

Live hearts of embryos labelled at the stage 22HH at the conal limits (GI) and re-incubated until they reached stage 24-36HH were used to evidence the apoptotic pattern in the embryonic outflow during cardiac septation (n = 5 at each stage). The hearts were isolated in 0.01 M phosphate-buffered saline (PBS, pH 7.4, 8 mM Na_2_HPO_4_, 2 mM KH_2_PO_4_, 136 mM NaCl, 2.6 mM KCI) supplemented with the vital dye LysoTracker Red (50 nM, LTR). After a 30 minutes incubation at 37°C, the hearts were washed (PBS) and fixed for 4 hours (4% formaldehyde in PBS). After extensive washing (PBS), LysoTracker fluorescence was visualized using an epifluorescence stereomicroscope Lumar V12 (Carl Zeiss, Germany) using a rhodamine filter. Special emphasis was placed on the anatomical regions between the labels previously inserted in the conal limits. Specimens were photographed and processed to histological analysis. Serial transverse sections (5 μm) were obtained for careful analysis under a CONFOCAL LSM-780 NLO microscope (Carl Zeiss, Germany).

### Immunofluorescence

Representative transversal histological sections from GI embryonic hearts (Stages 24-36HH) were washed and permeabilized with Tween 20 (Sigma-Aldrich, USA) in PBS and treated with serum-free protein block (Dako, USA). Tissues sections were incubated with a mouse anti skeletal muscle myosin antibody (Santa Cruz Biotechnology USA, SC-32732) and secondary rabbit anti-mouse antibody conjugated to Alexa Fluor 488 (Thermo Fisher Scientific, USA). The sections were then observed under a CONFOCAL LSM 780 NLO (Carl Zeiss, Germany). Controls for immunofluorescence experiments included the use of the secondary antibody alone.

## Results

### Prospective fate of the myocardial wall of the conus

**Fig 2:**
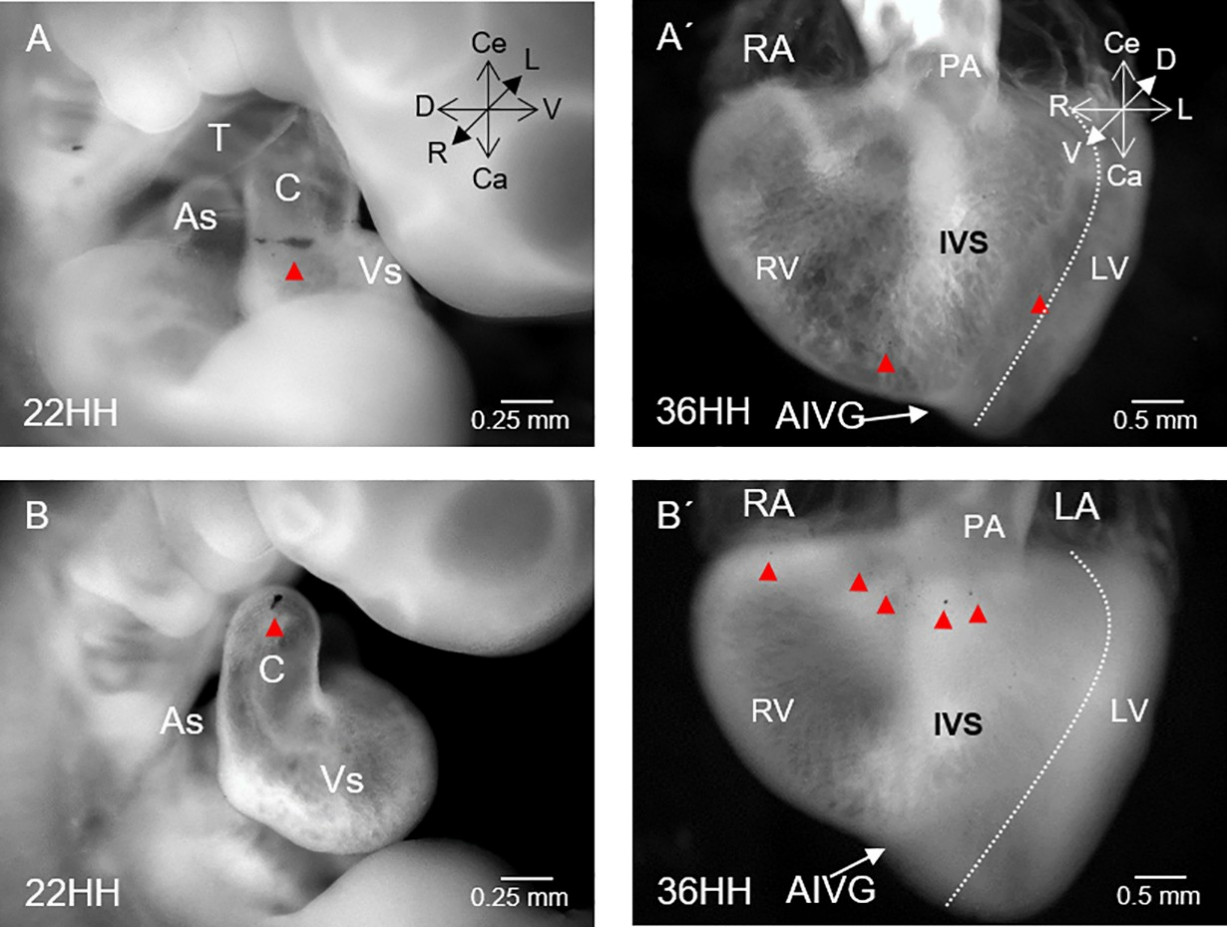
Prospective fate of the right conal wall (classically denoted anterior conus). (A) Representative images of embryonic heart at stage 22HH showing a black label inserted in the myocardium on the right surface of the cono-ventricular boundary (red arrowhead). (A’) Embryonic heart at stage 36HH. Visualization of small dark spots (red arrowheads) distributed in the myocardium of the apical ventricular region in relation to the anterior interventricular groove (AIVG). (B) Embryonic heart at stage 22HH with a black label inserted in the myocardium on the right surface of the conus-truncus border (red arrowheads). (B’) Embryonic heart at stage 36HH showing small dark spots (red arrowheads) distributed in the myocardium from the right region of the cardiac base to the subvalvular pulmonary region. The dotted white line indicates the limit of the right ventricular-free wall. Abbreviations: AIVG = anterior interventricular groove; As = atrial segment; C = conus; IVS = interventricular septum; LA = left atrium; LV = left ventricle; PA = pulmonary artery, RA = right atrium; RV = right ventricle; T = truncus, Vs = ventricular segment.

To determine the prospective fate of the right conal wall (classically denoted anterior conus), plastic black labels were placed in the myocardium at each boundary of conus and traced up to the mature heart (Fig 2). Labels initially inserted at the right surface of the conus-ventricular limit (Fig 2A) turned counter-clockwise towards to the embryonic middle line and in the mature heart (St 36HH) were distributed in the myocardium of the apical ventricular region in relation to the anterior interventricular groove (compare A with A’ in Fig 2). In contrast, labels placed at the beginning at the conus-truncus border, at stage 36HH, were located across the entire length of the right region of the cardiac base to the level of the sub-valvular pulmonary region (compare B with B’ in Fig 2).

**Fig 3:**
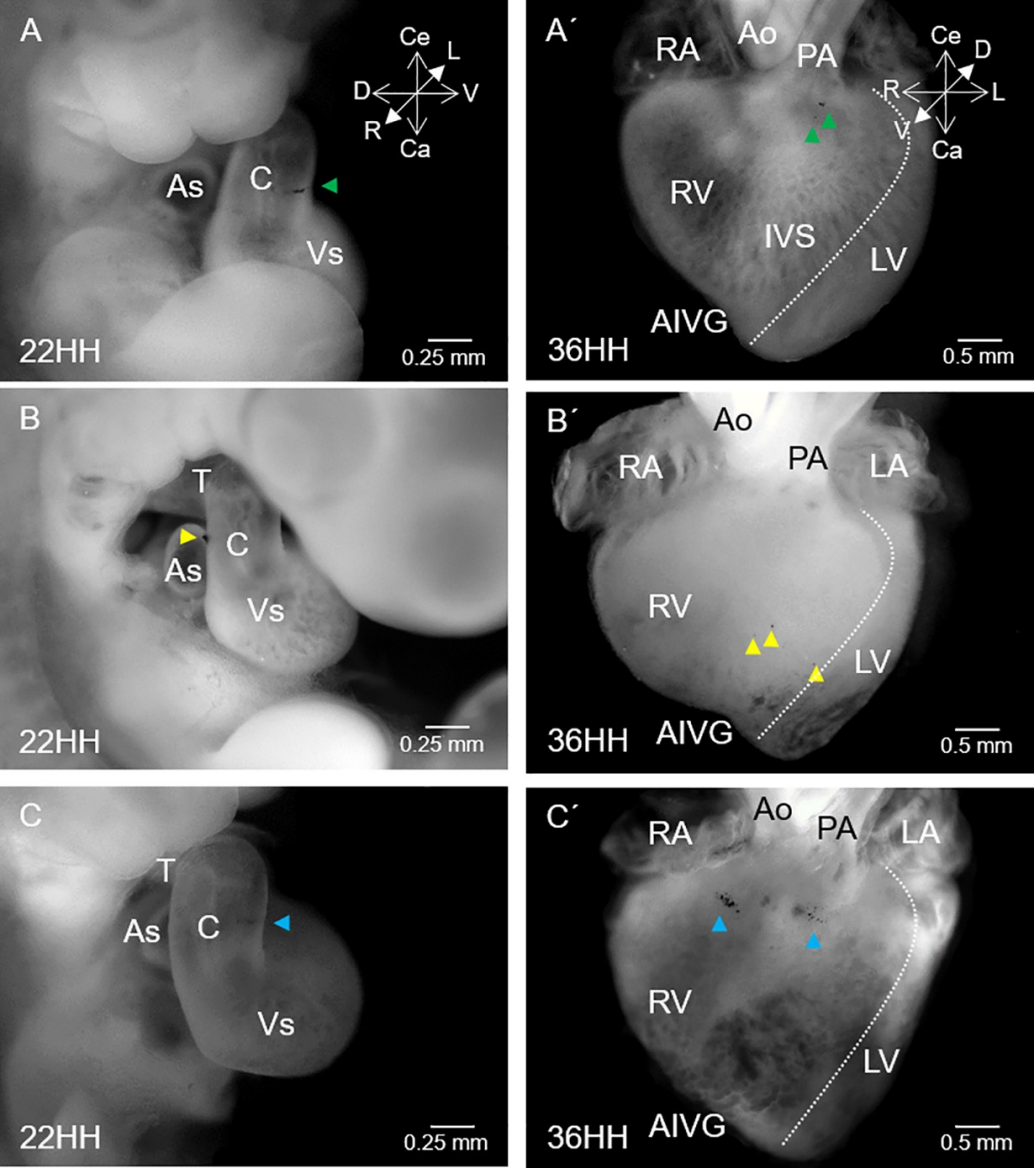
Prospective fate of the myocardium of the ventral, dorsal and left conal walls. (A) Embryonic heart at stage 22HH showing a black label inserted in the myocardium at the medial zone of the ventral conal wall (green arrowhead). (A’) Embryonic heart at stage 36HH with small dark granules (green arrowheads) distributed in the myocardium of the sub-valvular region of the pulmonary artery. (B) Embryonic heart at stage 22HH with a black label inserted in the myocardium of the dorsal conal wall (yellow arrowhead). (B’) Embryonic heart at stage 36HH showing small dark spots (yellow arrowheads) distributed in the myocardium along the middle third of the free wall of the right ventricle. (C) Embryonic heart at stage 22HH with a black label inserted in the myocardium of the medial zone of the left conal wall. (C’) Embryonic heart at stage 36HH. Visualization of some small dark spots (blue arrowheads) distributed in the myocardium between the acute border of the heart and the pulmonary infundibulum. The dotted white line indicates the limit of the right ventricular-free wall. Abbreviations: AIVG = anterior interventricular groove; Ao = aorta; As = atrial segment; C = conus; IVS = interventricular septum; LA = left atrium; LV = left ventricle; PA = pulmonary artery, RA = right atrium; RV = right ventricle; T = truncus, Vs = ventricular segment.

The myocardium of the left, ventral and dorsal conal walls was distributed to different anatomical portions exclusive of the right ventricle. Specifically, labels inserted in the myocardium of the ventral conal wall (Fig 3A) consistently remained in the same plane, revealing a limited displacement during development and in the mature heart were distributed in the myocardium of the sub-valvular region of the pulmonary artery (Fig 3A’). Labels inserted at the beginning in the myocardium of the dorsal wall of the conus (Fig 3B) turned counter-clockwise to the embryonic middle line, and labels in the mature heart were distributed in the myocardium along the middle third of the free wall of the right ventricle (Fig 3B’). In contrast, the labels placed at the beginning in the myocardium of the left conal wall (Fig 3C) showed an irregular behaviour. In almost all the hearts (90%), the labels gradually shifted counter-clockwise over time, and at the end of the experiment (Stage 36HH), they were distributed in the ventricular myocardium from the acute border of the heart to the middle portion of the pulmonary infundibulum (Fig 3C’). In some hearts (9%), the labels shifted clockwise and were distributed in a small portion corresponding to the left wall of the outflow tract of the right ventricle. Only 1% of the hearts showed an initial label in the myocardium of the left conal wall, in which the label was fragmented into two pieces. Each piece of the label was shifted in the opposite direction, and in the mature heart, one label appeared in the myocardium between the acute border of the heart and the pulmonary infundibulum. The other piece of label was found in the left wall of the right ventricular outflow tract. It is important to mention that labels inserted in the left, ventral and dorsal conal walls at the level of the conus-truncus border, in the mature heart were also distributed in the RV myocardium but in a more superior position than those inserted in the medial zone of the conus.

### Morphometric analysis

**Fig 4:**
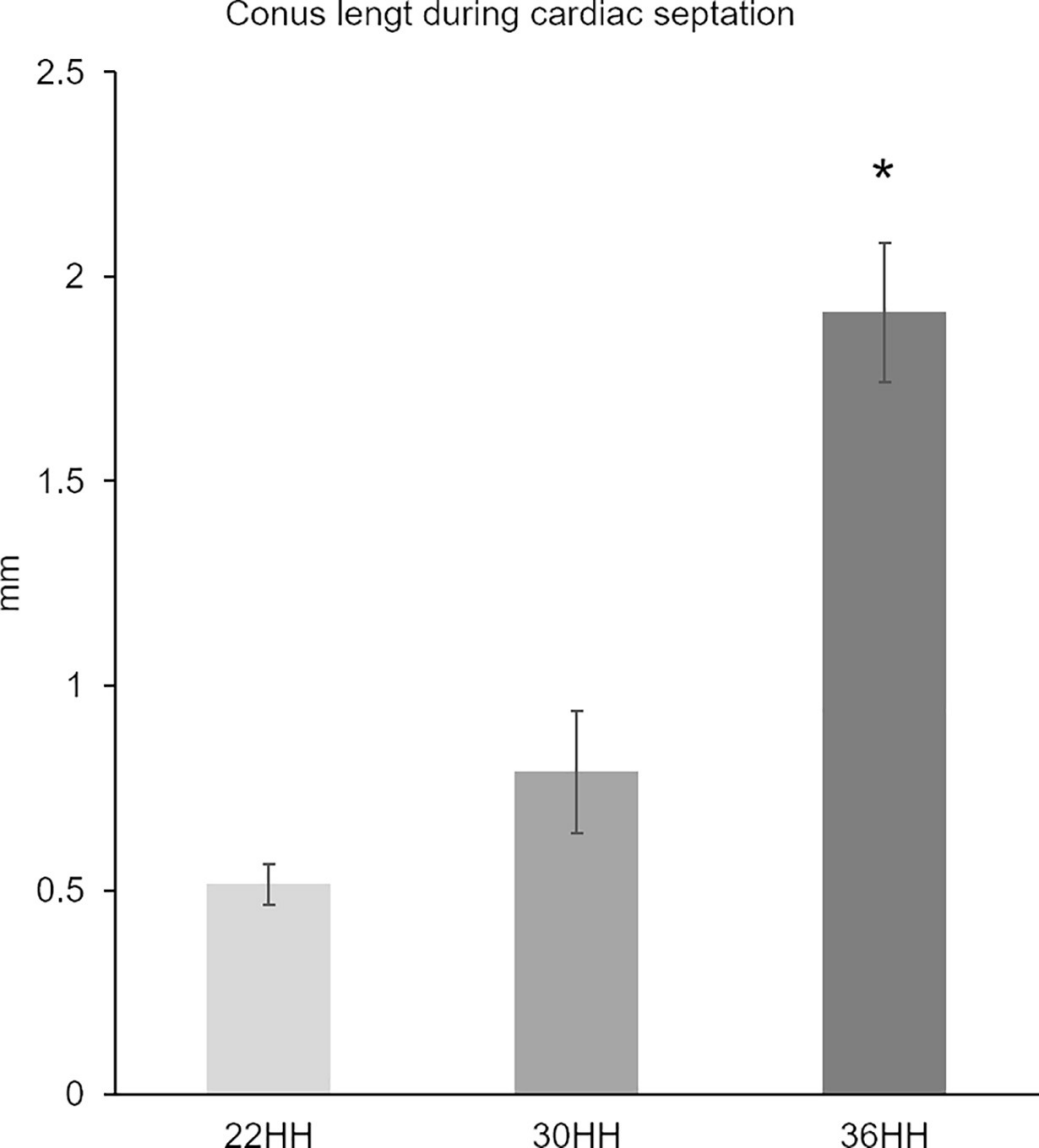
Increase in conus length during cardiac septation. Increase in conus length during cardiac septation was statistically significant between stages 22–36HH. Data are expressed as means (standard deviation) using *p ≤ 0.01 for comparisons.

In agreement with the results of the selective labelling experiments, the conus length registered during cardiac septation showed a differential growth of the conus during its transformation into mature structures. We observed that the conus length at stage 22HH was 0.51 mm (sd ± 0.049), at stage 30HH 0.78 mm (sd ± 0.150) and at stage 36HH, it had increased to 1.930 mm (sd ± 0.170). Between stages 22 to 36HH, the increase in conus length was statistically significant (Fig 4).

### Conus remodelling during cardiac septation

**Fig 5:**
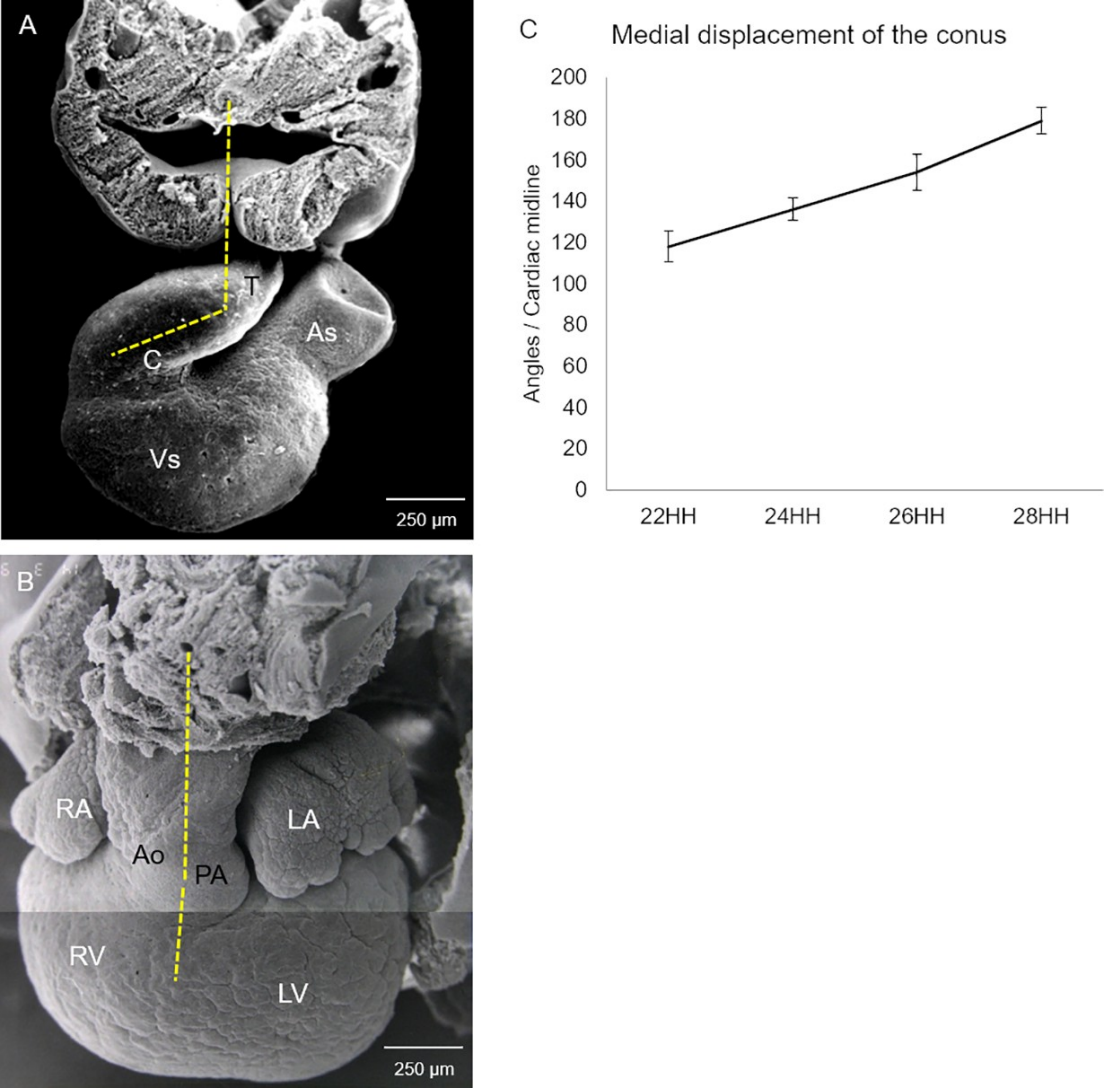
Conus gradual medial displacement during cardiac septation. Frontal views of the embryonic heart. (A) Stage 22HH visualization of the right extra cardiac initial position of the conus. (B) Stage 28HH visualization of the conus completely integrated into the right ventricle, occupying a ventral inferior position. (C) Graphic. Data are expressed as means (standard deviation) using *p ≤ 0.01 for comparisons. Abbreviations: Ao = developing aorta; As = atrial segment; C = conus; LA = left atrium; LV = left ventricle; PA = developing pulmonary artery, RA = right atrium; RV = right ventricle; T = truncus, Vs = ventricular segment.

To investigate how the conal myocardium remodels and moves from its originally right position to a ventral position in the mature heart, topologic and histological changes in the conus and AV canal during heart septation were registered. The results obtained in the captured images from GI hearts showed that angle from the middle line of the embryo and the sagittal line drawn up throughout the developing conus gradually increased. At stage 22HH, it had an opening of 120°, and it increased to 140° at stage 24HH, 160° at stage 26HH and 180° at stage 28HH (Fig 4C). Scanning electron microscopic images of embryonic hearts in the frontal view showed that the conus was completely integrated into the right ventricle at stage 28HH, occupying a ventral position from below the valvular floors of the developing pulmonary artery and aorta (compare A with B in Fig 5).

**Fig 6:**
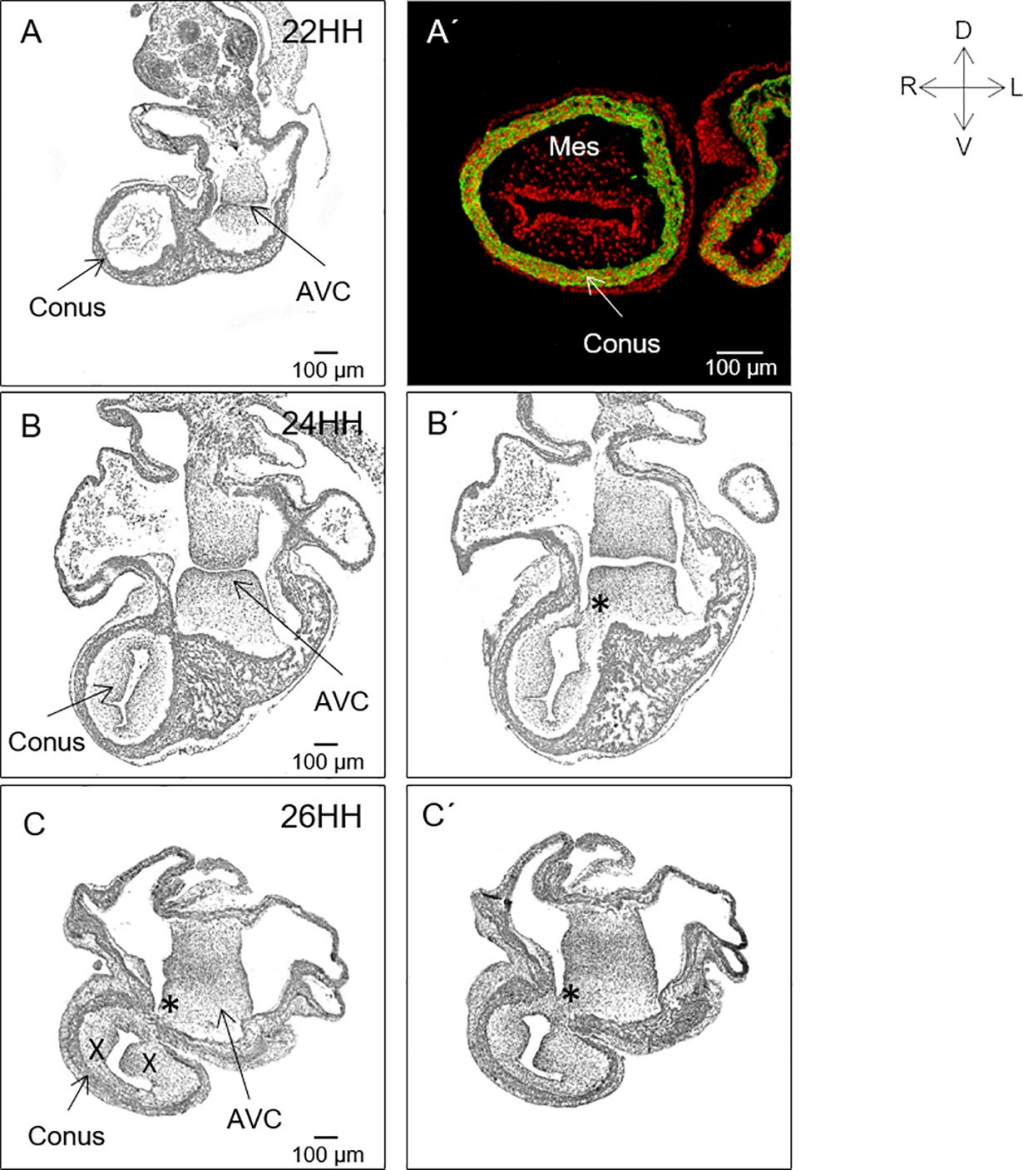
Conal myocardium and mesenchymal remodelling. Adjacent cephalo-caudal sections of the embryonic heart from stages 22HH to 26HH. (A) Stage 22HH. The lumen of the conus and the AV canal are side by side. (A’) Immunolocalization of the skeletal muscle myosin (green). Observe the tubular structure of the conal myocardium, the homogeneously distributed mesenchymal tissue (Mes), and the endocardium (red nucleus). (B, B’) Stage 24HH. The conus is in a slightly left and ventral position with respect to the AV canal. (B’). A small myocardial notch (*) is observed at the left-dorsal conal wall. (C, C’). Stage 26HH. Observe the loss of continuity at the thinned left-dorsal myocardial conal wall (*) and the incipient endocardial conal crest (X). Abbreviations: AVC = atrioventricular canal; Mes = mesenchymal tissue.

Serial histological sections of the embryonic hearts in the transverse plane showed that at stage 22HH, the lumen of the conus and the AV canal were in a contralateral position (Fig 6A). At this stage, the conus had a tubular structure formed by a thin layer of continuous myocardium (corroborated by the immunolocalization of skeletal muscle myosin) and was internally lined by endocardium (Fig 6A and 6A’). Inside the conus, we observed a homogeneous distribution of mesenchymal tissue formed by an abundant extracellular matrix with some fibroblast-type cells but without an apparent bulge arrangement (Fig 6A). At stage 24HH, the conus had moved from a right position to a slightly left and ventral position while the AV canal had moved from a left to a right position (Fig 6B). In addition, in a histological section closer to the ventricular segment, a small notch was observed in the myocardium of the left-dorsal wall of the conus that marked the beginning of the loss of continuity of the myocardium of that conal wall (Fig 6D). Mesenchymal tissue of the conus was still observed as a continuous layer, without signs of any incipient conal crest. The cushions of the AV canal and the mesenchymal tissue of the conus were more voluminous, with a larger cell population than at stage 22HH (Fig 6B and 6B’). At stage 26HH, the conus had been displaced to the left, and the AV canal was moved to the right (compare A, A’ with C, C’ in Fig 6). The myocardium of the ventral conal wall had thickened, but a small portion of myocardium at the left-dorsal wall had thinned, and in the zone adjacent to the cushions of the AV canal, the conal myocardium was losing continuity. Analysis of subsequent histological sections showed an increase in the conal myocardium opening in the cephalo-caudal direction (compare C with C’ in Fig 6). Additionally, the mesenchymal tissue of the conus began to show signs of separation in two incipient conal crests. At this stage (26HH), the ventro-superior and dorso-inferior cushion of the AV canal were already fused. The conal crest and the fused AV cushions mesenchyme were in contact at the opening of the myocardial conal wall (Fig 6C and 6C’).

**Fig 7:**
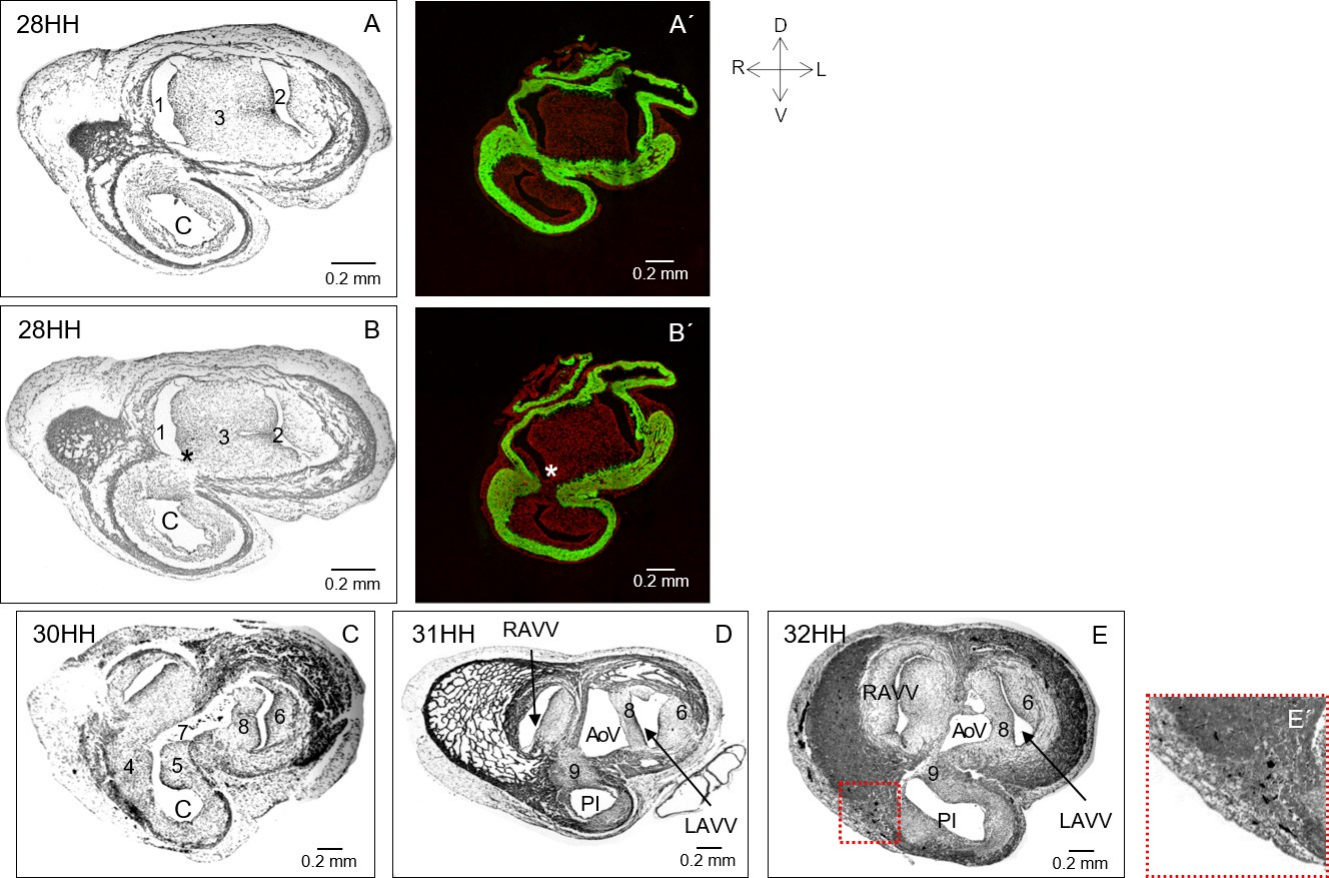
Pulmonary vestibule and the aortic infundibulum develop from different embryonic components. (A, B). Adjacent cephalo-caudal histological sections of the embryonic heart at stage 28HH. Observe a wider opening zone at the originally left-dorsal myocardial conal wall (*). The voluminous mesenchymal structure separating the incipient right (1) and left (2) atrioventricular orifices corresponding to the ventro-superior and the dorso-inferior cushions of the AV canal during the fusion process (3). (A’, B’) Skeletal muscle myosin immunolocalization (green) corroborating the left-dorsal myocardial conal wall loss of continuity (*). (C) Stage 30HH. Visualization of the well-developed dextrodorsal (4) and sinistroventral (5) crests within the conus (C). The incipient mural leaflet of the left AV valve (6) is also evident. Note the elongated canal (7) bordered by the dorsal surface of the conal crest (4 and 5) and the right surface of the incipient anteroseptal leaflet of the left AV valve (8). (D, E). Stage 31 and 32HH. Mature heart showing the fused conal crests forming the immature supraventricular crest (9). The single conal conduct corresponds to the pulmonary infundibulum (PI). The aortic vestibule (AoV) behind the developing anteroseptal leaflet of the left AV valve (8) is well developed. E’. Amplification of the red box in E. Observe traces from the label as some fine charcoal granules immersed in the myocardium of the pulmonary infundibulum (40X). Abbreviations: RAVV = Right atrioventricular valve; LAVV = Left atrioventricular valve.

At stage 28HH, the myocardium of the left-dorsal wall of the conus continued losing tubular continuity, thus showing a wider opening zone in an dorso-ventral direction (compare A with B in Fig 7). The loss of contact of the myocardium at the conal left-dorsal wall was corroborated by skeletal muscle myosin immunolocation (Fig 7A’ and 7B’). Contact between the mesenchymal tissue of the conal crest and that of the fused cushions of the AV canal had increased, without leaving any signs of separation between them. The right and left cushions of the AV canal were evident (Fig 7A and 7B). At this stage (28 HH), the recently fused AV cushions had formed a voluminous mesenchymal structure that separated the incipient right and left AV orifices. At stage 30HH, the myocardium of the conus had been transformed from a closed tubular structure in the form of a letter "O" to acquire the shape of a letter “U” (Fig 6C). The originally incipient dextrodorsal and sinistroventral conal crests had increased in volume and were fused at the level of conus-truncus border but not at the level of the AV valves (Fig 7C). Likewise, the left cushion of AV canal had been remodelled to form a mesenchymal structure that resembled the mural leaflet of the left AV valve, while the dorso-inferior and ventro-superior AV cushions in the process of fusion were forming an incipient anteroseptal leaflet of the left AV valve (Fig 7C). In this heart (stage 30HH), an elongated canal was evident, the edges of which were represented by the dorsal surface of the conal crest (in the fusion process), the right surface of the incipient anteroseptal leaflet of the left AV valve and the interventricular foramen (Fig 7C). Later, at stage 31HH, the mesenchymal conal crests were fused, and a single pulmonary conal conduct was formed (Fig 7D). The elongated canal bordered by the conal crests, the right surface of the anteroseptal leaflet of the left AV valve and the interventricular septum were transformed in the aortic vestibule (Fig 7D). At stage 32HH, the conal myocardium had acquired the shape of a myocardial cell sheet (Fig 7E). The already fused conal crest formed a structure of mixed histological constitution (mesenchymal and myocardial tissues) that separated the right ventricular inlet from the completely myocardial pulmonary infundibulum. Likewise, the aortic vestibule bordered by the fibrous anteroseptal leaflet of the left AV valve and the myocardial interventricular septum was completely developed (Fig 7E).

### Apoptotic pattern at the embryonic outflow

**Fig 8:**
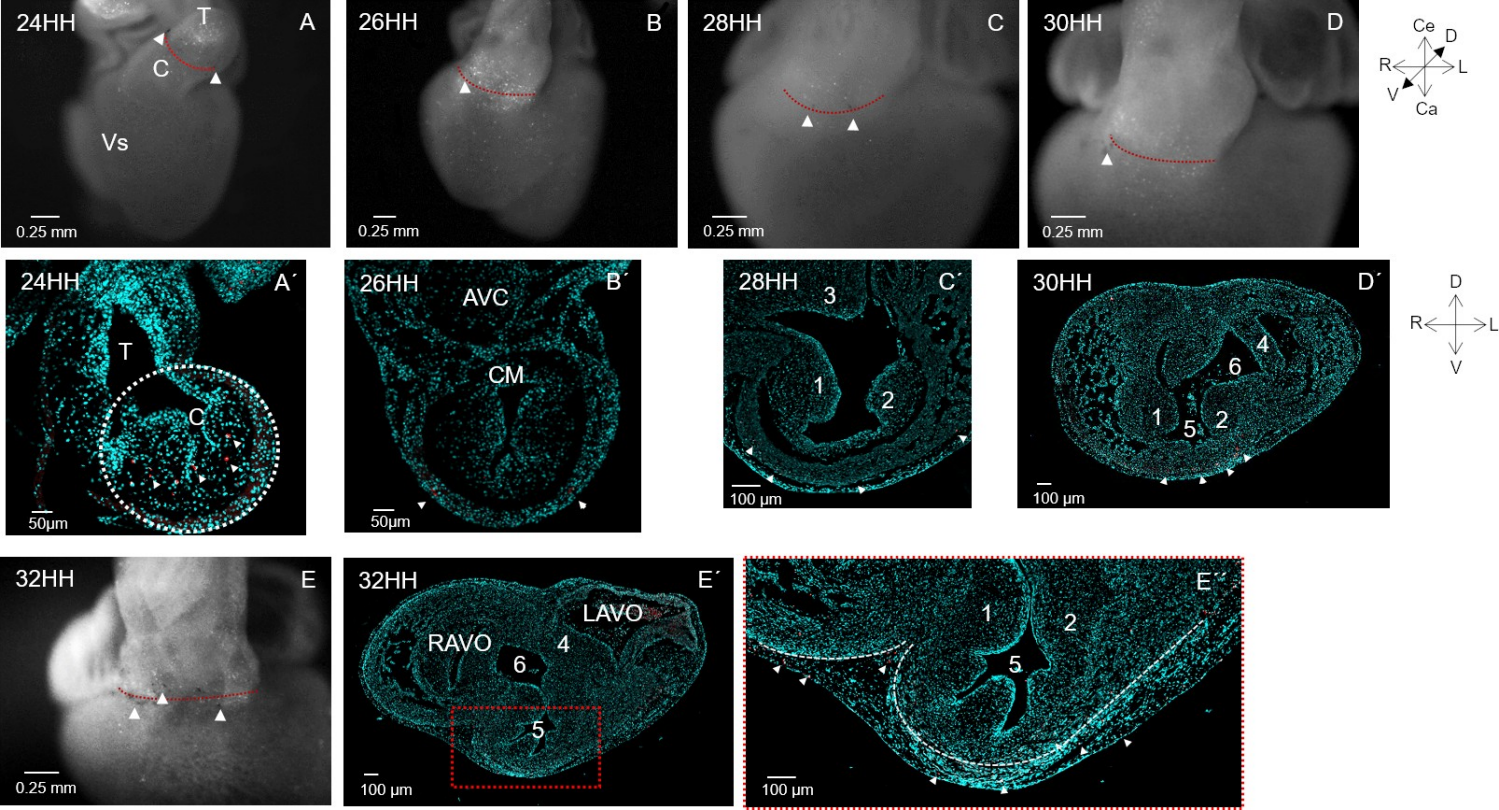
Apoptotic pattern at the embryonic outflow. (A–E) Representative images of embryonic hearts (stages 24-32HH) that have been previously labelled at the conus truncus limit and treated with LysoTracker Red. Visualization of positive apoptotic cells, mainly at the level of the previously labelled conus-truncus border, dotted red line. (A’- E”) Histological transversal sections of embryonic hearts treated with LysoTracker Red at the conus level. In this case, the apoptotic cells correspond to the red dots with white arrowheads. (A’) Stage 24HH, apoptotic cells were observed in the conal mesenchyme. (B’) Stage 26HH, some apoptotic cells were observed in the myocardium of the ventral conal wall. No apoptotic cells were detected in the myocardium of the thinner left-dorsal conal wall between the conal mesenchyme (CM) and the atrioventricular cushions (AVC). (C’) Stage 28HH. Observe few apoptotic cells immersed in the myocardium of the ventral conal wall. (D’- E”) Stages 30 and 32HH. Visualization of apoptotic cells principally in the epicardium. Abbreviations: LAVV = left atrioventricular oprifice; RAVV = right atrioventricular orifice. 1. Dextrodorsal conal ridge; 2. sinistroventral conal ridge; 3. fused atrioventricular cushions; 4. septal leaflet of the mitral valve; 5. pulmonary infundibulum; 6. aortic vestibulum.

To investigate whether the conus is affected by apoptosis, as is frequently affirmed, and to determine whether the loss of continuity of the left-dorsal conal wall depended on this process, the spatio-temporal pattern of apoptotic cells at the conus during cardiac septation was registered. In all the stages studied (22-32HH), positive apoptotic cells were observed predominantly at the conus-truncus border, identified by the previously placed plastic labels; only a few apoptotic cells were detected at the conus (Fig 8A–8D). Transverse histological sections of the embryonic heart allowed us to distinguish that the apoptotic cells at stage 24HH corresponded exclusively to the mesenchymal tissue of the endocardial conal crest; no traces of apoptosis were observed in the conal myocardium (Fig 8A’). In contrast, at stage 26HH, some apoptotic cells were observed in the myocardium of the ventral conal wall (Fig 8B’). Later, at stage 28HH, several apoptotic cells were detected dispersed in the myocardium of the originally definite right conal wall compared to the apoptotic cells at the conus-truncus limit (Fig 8C’). At stage 30HH, apoptotic cells were registered at the myocardium of the originally left conal wall. Between stages 30-32HH, the apoptotic signal was observed below the valvular aortic and pulmonary floors (Fig 8D and 8E). At these stages, the histological sections showed fewer apoptotic cells in the conal myocardium, but a large number of positive apoptotic cells were identified in the epicardium (Fig 8D’, 8E’ and 8E”). Surprisingly, no apoptotic cells were found in area adjacent to the conal myocardium opening (Fig 8B’).

## Discussion

The embryonic components and morphogenetic processes involved not only in the development of the intracardiac pulmonary infundibulum and aortic vestibule, but also in the formation of the extracardiac trunks of the great arteries are controversial. In our opinion, the discrepancies are caused, in part, because many reports describe results without specifying the region of the embryonic outflow tract (proximal or conus), intermediate (or truncus) or distal (aortic sac) are involved. This information is a key point because although initially the truncus and conus are morphologically and histologically similar, each has a different anatomical manifestation in the postnatal heart (6–17). Additionally, although it is generally stated that the conus participates in the development of the pulmonary infundibulum and aortic vestibule [6, 7, 23, 24], some researches involve the ventro-superior cushion of the AV canal in the development of the aortic vestibule [26, 27]. Therefore, our goal was to investigate, by selective *in vivo* labelling, the prospective fate of the myocardium of the right, left, ventral and dorsal conal walls. The changes in the mesenchymal tissue of the conus and AV cushions and the role of apoptosis in conus transformation into mature structures were also explored.

### Origin and fate of the conal myocardium

Recently, the concept of the FHF represented by the straight heart tube and the second heart field (SHF) originating later and gradually converging into the straight heart tube during the torsion and looping process has been emerging [3–5]. This new finding has led to controversy about the prospective fate of the straight heart tube. On the one hand, it was accepted that the precursor cells of the RV in the chicken embryo are already present in the straight heart tube [36]. Furthermore, it has been pointed out that the conus appears in the “C” shaped looped heart and completes development in the advanced looped heart (stage 22HH) to form both ventricular outflow tracts [24]. In this case, it is inferred that the RV would derive from the FHF, while the conus would develop from the SHF. Findings in the mouse embryo are discordant with these statements. Through genetic tracking, it has been demonstrated that the FHF gives rise to the myocardium of the LV and the atria [5]. Likewise, the SHF has been identified as the cellular source from which the conus and truncus develop in both chicken and mouse embryos [3, 4, 30, 31, 32, 37]. *Our in vivo* labelling in the chick embryo heart of the conal walls (stage 22HH to 36HH) show that the conal myocardium is gradually distributed into different regions of the RV free wall, from the apex to the cardiac base, below the arterial valves (Figs 2, 3 and 8B). Our morphometric and topological studies that show a length increase in the conus and its gradual movement from its original right position to a definitive ventral position (Fig 5C) agree with our *in vivo* labelling results. These findings, confirm that in the chick embryo the conus completes development at stage 22HH [24], and indicate that the RV myocardium both in birds and in mammals, is not formed by cell populations present at the straight heart tube as indicated in pioneering *in vivo* labelling studies in the chick embryo heart [36], really the RV myocardium is developed from the region of the SHF that represents only the conus at stage 22HH.

### Conal remodelling during cardiac septation

**Fig 9:**
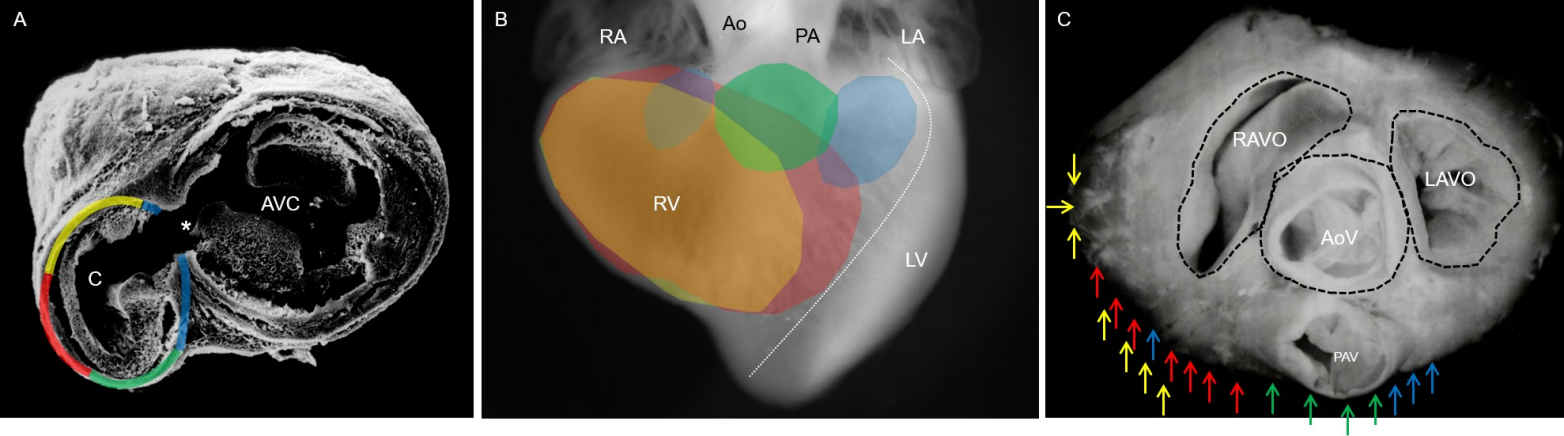
Fate map of the conal walls based on the labelling experiments. (A) Micrograph obtained using a scanning electron microscope of a 26HH chicken embryo heart dissection. The results show the partial displacement of the conus on the way to its final ventral position and the discontinuity of the dorsal-left wall conal myocardium (*). (B) Frontal view of the chicken embryo heart at stage 36HH, depicting the myocardial conal wall distribution exclusively throughout the right ventricle (RV). The white dotted line marks the edge of the free wall of the right ventricle. (C). Chicken postnatal heart dissection showing differential fate of each of the conal walls in the right ventricle below the arterial and atrioventricular valves. The black dotted line denotes the fibrous skeleton where the pulmonary valvular ring (PA) is excluded.

It is universally accepted that ventricular outflow tracts develop via the fusion of two mesenchymal crests in the lumen centre of the conus to originate two conduits: the anterior or pulmonary and the posterior or aortic conuses [6, 7, 23, 24, 38–40]. In contrast, in absolute discrepancy with this idea, we found that the conal myocardium participated in formation of the RV free wall, including its outflow. This proposal questions the existence of the posterior conus. Thus, to understand how the conal myocardium changed to be distributed along the RV ventral wall and the fate of the supposed posterior conus, serial histological sections of the embryonic heart in the transverse plane were analysed (stages 22-34HH). We confirmed our *in vivo* labeling and morphometric findings (Figs 5–7). However, when analysing the conal and AV canal mesenchymal tissue changes, we observed important events that had not yet been reported in the development of the conus. First, although it is common to describe the presence of two mesenchymal endocardial crests inside the conus from stage 22HH, we actually observed a gradual development of the conal mesenchyme. It was initially scarce and homogeneously distributed between the myocardium and endocardium (22HH). Later, at stage 24HH, the conal mesenchyme maintained a homogenous distribution but increased in volume. Between stages 26-28HH the conal ridges had developed in the dorsal zone of the conal lumen. These results support the idea that the mesenchymal tissue that internally covers the conus at earlier stages has a valvular function to prevent blood return to the ventricular segment, but it does not participate in conus septation [41– 43]. Additionally, we found that at stage 22HH the conus had a tubular structure, which was externally covered by a thin layer of continuous myocardium and internally lined by endocardium with a thin extracellular matrix (Fig 6A and 6A’). In no histological section did we observe the presence of two well formed conal crests between stages 22-24HH. Between stages 23-28HH, the myocardium of the left dorsal conal wall lost continuity. The free borders of the conal myocardium gradually separated from each other until they formed a myocardial cell sheet (compare Fig 6B’ and 6C’ with Fig 7, Fig 9A). The absence of apoptosis in the opening region of the myocardium of the left dorsal conal wall indicated that the loss of continuity of that conal wall occurred independently of programmed cell death. Additionally, the progressive opening of the left-dorsal conal wall observed in the histological sections agreed with our *in vivo* labelling of the same conal wall (Figs 2 and 3). Taken together, these findings provide unequivocal evidence that the conus is the embryonic precursor of the RV, including the pulmonary infundibulum (Fig 9B). Likewise, although Rana et al [25] reported that the RV free wall forms from the “outflow myocardium”, we based on our *in vivo* labelling results; conclude that only the conus but not the truncus participates in this process.

### Apoptosis and conus development

Massive myocardial apoptosis is a morphogenetic process that is proposed to explain the longitudinal reduction of the embryonic outflow [28, 29, 44]. However, due to the impossibility of distinguishing the conal and truncal myocardium by viral transfection, Watanabe and colleagues concluded that both embryonic structures disappear almost completely, causing a longitudinal shortening of the embryonic outflow tract [28, 29]. In contrast, by selective labelling, we distinguished the conus from the truncus myocardium and found a spatiotemporal apoptotic pattern concordant with that described by Cheng in 2002 [45]. As Cheng we found that during development, the truncus was mostly affected by apoptosis and that apoptotic focus began to appear by stage 26HH in small clusters at the myocardial conus-truncus border. (Fig 8A–8E). We also observed that apoptosis in the conal domain shifted sequentially, starting in the conal mesenchyme (Fig 8A’), later occurring in the lateral myocardium of the developing conus (Fig 8B’–8D’), and finally being detected in the epicardium (Fig 8E”). These results suggest that apoptosis does not participate in conal resorption but contributes to the fine remodelling that allows conal transformation in a large part of the RV free wall. We can also suppose that in the truncus apoptosis is linked to the arterial valves remodeling.

### Conus septation

Septation of the conus occurs in the chicken embryo between stages 26-32HH. In this context, it is universally known that during this process, the conal crests fuse in the centre of the conal lumen to form the aortic and pulmonary conuses [6, 7, 23, 24, 38–40]. However, when analysing the development of the conal mesenchyme, we simultaneously found that the myocardium of the left dorsal conal wall was opening between stages 26-28HH, and two well developed conal crests had developed in the dorsal zone of the conus lumen (Figs 6C and 6C’, 7A and 7C). Later, between stages 30-32HH, the conal crests increased in volume and cellularity, approaching each other (30HH) to finally fuse (31HH) on the dorsal surface, but not at the centre of the conal lumen, as has been consistently clammed (Fig 7C). Interestingly, between stages 31 to 32HH, we observed that when the conal crests were fused, two ducts were not formed, but rather the fused crests formed a mesenchymal structure that separated the incipient inflow and outflow tracts of the RV (Fig 7D and 7E). These results, in addition to refuting the existence of the posterior conus, agree with previous findings obtained by *in vivo* labelling of the chick embryo heart showing that the supraventricular crest develops from the fused conal crests [46]. The initially mesenchymal supraventricular crest is gradually transformed into a myocardial structure by “myocardialization”. At present, there is no consensus regarding the characterization of this process. It is possible that the mesenchyme could serve as a scaffold for cardiomyocyte migration from the ventricular walls, the AV canal and / or the interventricular septum. Alternatively, mesenchymal cells could be transformed in myocardiocytes.

### Importance of AV cushions in the development of the aortic vestibule

In addition to separating the primitive inlet in two conducts, the AV cushions participate in AV valve development. In the eighties, a group of researchers *in vivo* labelled the ventral (superior) cushion of the AV canal in the chick embryo heart (stage 22HH). In the mature heart, the labels were found, surprisingly, in both components of the aortic infundibulum, i.e., the free region of the anteroseptal leaflet of the left AV valve and the muscular region of the interventricular septum separating the aortic vestibule and the pulmonary infundibulum [26, 27]. These researchers could not explain the role of the posterior conus, the supposed precursor of the aortic vestibule. However, using transverse histological sections, we could observe the conal crest formation and a volume increase as well as more cellularity in the AV cushions at stage 26HH, which led to the fusion of both AV cushions (Fig 6C and 6C’). Almost immediately, the cushion mesenchyme was remodelled, thinned and flattened, thus acquiring the shape of a thick arch (Fig 7A and 7C). Later, at stage 30HH, the voluminous conal crests were almost in contact. In this heart, the concave edge of the mesenchymal arch resulting from the fusion of the ventral (superior) and dorsal (inferior) AV cushions had acquired the shape of an incipient septal leaflet of the left AV valve (Fig 7C). The right surface of the incipient septal leaflet of the left AV valve represented the edge of the aortic vestibule, which in this stage was observed as a long narrow canal that was continuous with the pulmonary infundibulum because the conal ridges had not yet fused (Fig 7C). Later, at stage 31-32HH, the already fused conal crests had the location and appearance of the partially muscularized supraventricular crest. The septal leaflet of the left AV valve was thinner, maintained a fibrous structure and delimited the individualized LV outflow (Fig 7D and 7E). These results further confirmed that when fused, the conal crests formed the myocardial supraventricular crest [46], leading to the conclusion that the ventricular outflow tracts originated from different embryonic components. The distal part of the conus with the fused conal crest participates in the development of the completely myocardial pulmonary infundibulum, while the predominantly mesenchymal aortic vestibule is formed from the ventro-superior cushion of the AV canal, as previously evidenced by *in vivo* labelling [26, 27]. Additionally, based on our results for the differential development of the conal and AV cushions mesenchyme, and new evidence of a differential endocardium to mesenchyme transformation molecular regulation pathway [47], we suggest the use of the classic conal crest and AV cushions nomenclature.

Our findings refuting the existence of the posterior conus and showing evidence of a distinct embryonic origin for both outflow tracts are in agreement with the anatomic description of the cardiac fibrous skeleton (Fig 9C) that includes the mitral, tricuspid and aortic valves with the mitral-aortic fibrous continuity but excludes the pulmonary valve [48]. Additionally, these same results allow us to speculate that persistence of the conus as a tubular structure would lead to the development of a univentricular heart, defined by Anderson as a heart with absence of the posterior interventricular septum [49].

The new information obtained in the present investigation, provides a frame of reference for the molecular approaches of the origin of the pulmonary infundibulum and the aortic vestibule. Additionally, it provides solid embryological bases for the improved diagnosis and surgical treatment of the congenital defects that affect these anatomical structures of the heart.
